# Nrf2 activity as a potential biomarker for the pan-epigenetic anticancer agent, RRx-001

**DOI:** 10.18632/oncotarget.4249

**Published:** 2015-06-04

**Authors:** Shoucheng Ning, Thillai Veerapazham Sekar, Jan Scicinski, Bryan Oronsky, Donna M. Peehl, Susan J. Knox, Ramasamy Paulmurugan

**Affiliations:** ^1^ Department of Radiation Oncology Stanford University Medical Center Stanford, CA 94305, USA; ^2^ Department of Radiology Stanford University Medical Center Stanford, CA 94304, USA; ^3^ Department of Urology Stanford University Medical Center Stanford, CA 94305, USA; ^4^ EpicentRx, Inc., Mountain View, CA 94041, USA

**Keywords:** Nrf2, ARE, biomarker, oxidative stress, cancer

## Abstract

Nuclear factor erythroid 2-related factor 2 (Nrf2) is a master regulatory transcription factor that plays an important role in the antioxidant response pathway against anticancer drug-induced cytotoxic effects. RRx-001 is a new anticancer agent that generates reactive oxygen and nitrogen species, and leads to epigenetic alterations in cancer cells. Here we report the RRx-001 mediated nuclear translocation of Nrf2 and the activation of expression of its downstream enzymes HO-1 and NQO1 in tumor cells. Inhibition of intrinsic Nrf2 expression by Nrf2-specific siRNA increased cell sensitivity to RRx-001. Molecular imaging of tumor cells co-expressing pARE-Firefly luciferase and pCMV-Renilla luciferase-mRFP *in vitro* and *in vivo* in mice revealed that RRx-001 significantly increased ARE-FLUC signal in cells in a dose- and time-dependent manner, suggesting that RRx-001 is an effective activator of the Nrf2-ARE signaling pathway. The pre-treatment level of ARE-FLUC signal in cells, reflecting basal activity of Nrf2, negatively correlated with the tumor response to RRx-001. The results support the concept that RRx-001 activates Nrf2-ARE antioxidant signaling pathways in tumor cells. Hence measurement of Nrf2-mediated activation of downstream target genes through ARE signaling may constitute a useful molecular biomarker for the early prediction of response to RRx-001 treatment, and thereby guide therapeutic decision-making.

## INTRODUCTION

Nuclear factor erythroid 2-related factor 2 (Nrf2) is a redox-sensitive master regulatory transcription factor that plays an important role in the antioxidant response pathway against chemotherapeutic drug-induced cytotoxic effects by oxidative stress [1]. Nrf2 in particular regulates the expression of antioxidant genes such as heme oxygenase 1 (HO-1), glutamate cysteine ligase catalytic subunit (GCLC), and NAD(P)H dehydrogenase quinone 1 (NQO-1), which neutralize intracellular accumulation of reactive oxygen species (ROS). Nrf2 protein level in cells is regulated by a cluster of inhibitory proteins, including Kelch-like ECH-associated protein 1 (Keap1) and Cullin 3 (Cul3) ubiquitin ligase. Keap1 represses Nrf2 activity by binding to its Neh2 domain and consequently promoting contact between Nrf2 and the Cul3/Rbx1 ubiquitin ligase complex, leading to ubiquitination and degradation of Nrf2 by proteasomes [2]. Under normal conditions, Keap1 retains Nrf2 in the cytoplasm and ubiquitinylates Nrf2 to maintain its cellular threshold. Under oxidative stress, the Nrf2-Keap1 interaction is disrupted by the modification of Keap1 at cysteine 151 and protein kinase C-mediated phosphorylation of Nrf2 at serine 40 [3–4]. These modifications allow the release of Nrf2 from Keap1, resulting in the translocation of Nrf2 from cytoplasm to nucleus, where Nrf2 heterodimerizes with small Maf or Jun proteins and binds to the antioxidant response element (ARE) in the upstream UTR of promoter regions and initiates transcription of antioxidant genes [5–6]. Many studies have reported that Nrf2 and its downstream target genes are overexpressed in cancer cells, providing a growth advantage and contributing to therapeutic resistance against chemotherapy and radiotherapy through activation of antioxidant genes and neutralization of ROS accumulation [7].

RRx-001 is an aerospace-derived anticancer agent with reactive nitrogen species (RNS)-generating chemistry that leads to epigenetic alterations, such as DNA methylation and histone acetylation in cancer cells [8–13]. RNS, a collective term that includes highly reactive species such as peroxynitrite (ONOO^−^), nitrogen dioxide radical (•NO_2_), and other nitrogen oxides, are formed when nitric oxide (NO), which is abundantly induced by RRx-001 under hypoxic conditions, reacts with superoxide anion (O_2_^−^). In turn, RNS regulate DNA methyltransferases and histone deacetylases. The broad-spectrum epigenetic modulator activity of RRx-001 leads to resensitization of chemo- and radio-resistant tumor cells to therapeutic intervention, and is a focus of several ongoing clinical trials [13, 14]. Cell growth arrest induced by RRx-001 correlates with increased ROS/RNS production. Inhibition of ROS generation by N-acetylcysteine attenuates the antiproliferative effects of RRx-001 [11]. These findings suggest a crucial role of ROS and RNS as effectors of RRx-001-induced pro-oxidant damage and epigenetic activity in cancer cells.

A Phase I clinical trial demonstrated encouraging therapeutic responses in a heavily pretreated, chemo- and radioresistant patient population with multiple types of cancers. RRx-001 monotherapy was well tolerated, with no dose-limiting toxicities [14]. Preliminary data from an ongoing randomized proof-of-concept Phase II study of RRx-001 vs. regorafenib suggest a positive trend in overall survival compared to regorafenib alone in patients with advanced colorectal cancer (Carter C. et al. Annals of Oncology. 2015; 26(suppl 2):ii4-ii5).

To date no predictive factor of response has been described for RRx-001. Herein, we report the results of molecular imaging studies *in vitro* and in mice bearing murine squamous cell carcinoma SCC VII tumors co-expressing ARE-Firefly luciferase reporter to measure ARE signaling while constitutive CMV-Renilla luciferase-mRFP fluorescent protein measures cell viability in response to treatment with RRx-001. The results suggest that Nrf2-ARE and its downstream gene expression may serve as a biomarker for predicting response of tumors to RRx-001 treatment, and to select cancer patients who would be most likely to respond to and benefit from RRx-001 therapy.

## RESULTS

### Endogenous Nrf2 expression in RRx-001-treated cells *in vitro* and *in vivo*

**Figure 1:**
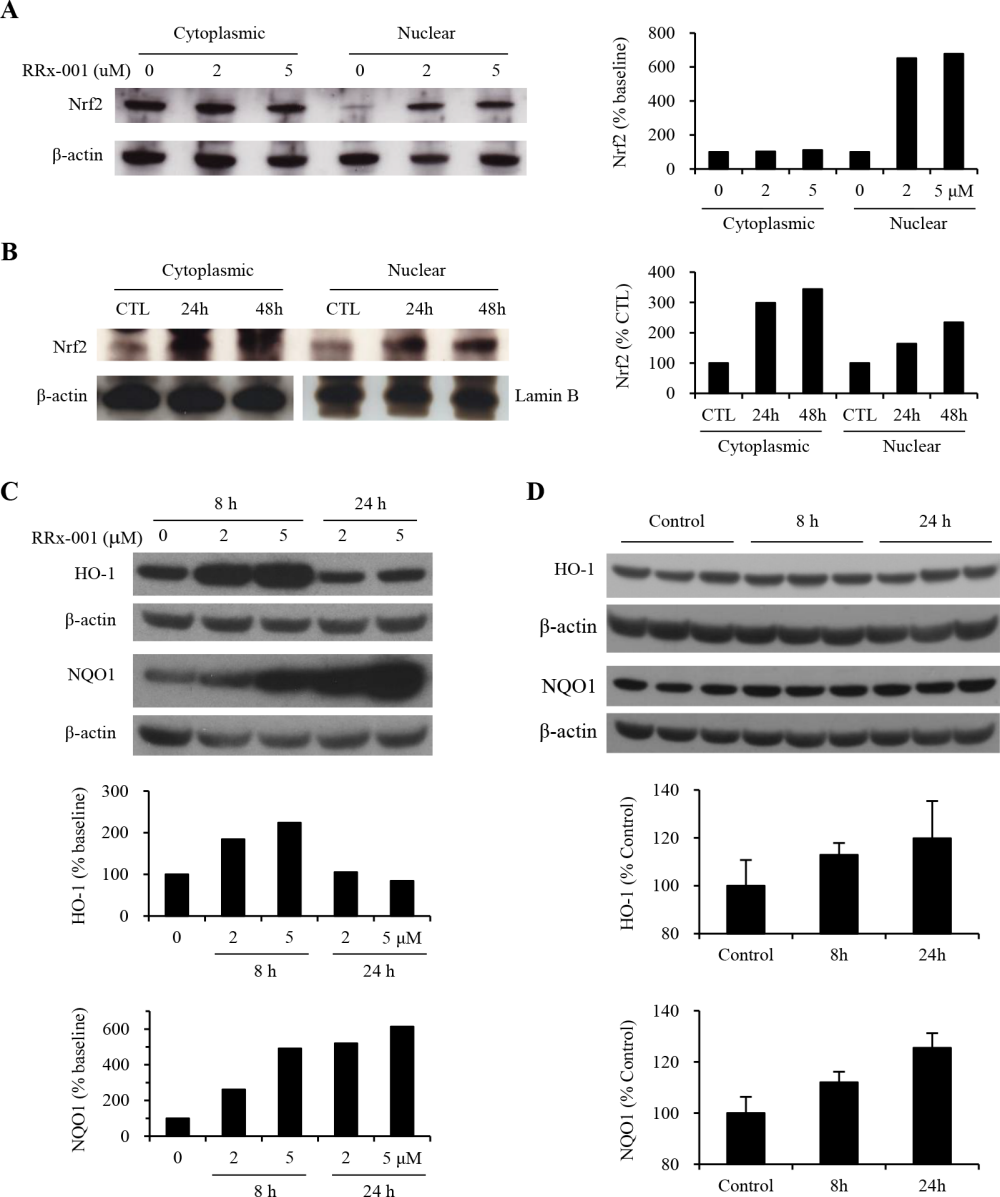
Nrf2, HO-1 and NQO1 expression in SCC VII tumor cells *in vitro* and *in vivo* **A.** Nuclear and cytoplasmic Nrf2 levels in SCC VII cells *in vitro*. **B.** Nrf2 in tumors from mice treated with a single dose of solvent (CTL) or 10 mg/kg of RRx-001, and tumor samples were collected at 24 h or 48 h after RRx-001 injection. **C.** HO-1 and NQO1 expression in cells *in vitro* treated with 0, 2, and 5 μM of RRx-001 for 8 h and 24 h. **D.** HO-1 and NQO1 in tumors from mice treated with one dose of solvent (Control) or 10 mg/kg of RRx-001 and tumors were collected 8 h or 24 h post-treatment. There are three tumors per group presented in the Western blots, and the average levels of HO-1 and NQO1 of 6 tumors in each group were presented in the plots.

Intracellular oxidative stress disrupts Nrf2-Keap1 binding, resulting in the release and translocation of Nrf2 from cytoplasm to nucleus, where Nrf2 binds to the ARE sequence in the 5′-UTR of antioxidant genes and initiates transcription (5–6). To investigate whether RRx-001-generated ROS and RNS activate endogenous Nrf2 in tumor cells, SCC VII cells were treated with 2 μM or 5 μM RRx-001 for 24 hours (h). Cells were lysed and cytoplasmic and nuclear proteins were isolated for immunoblot analysis. Results showed that the intensity of the cytoplasmic Nrf2 protein bands was not significantly different in cells with or without treatment of RRx-001 (Figure 1A). However, treatment with 2 or 5 μM RRx-001 caused a 6-fold increase in the amount of nuclear Nrf2 protein compared to the baseline (0 μM RRx-001), indicating that RRx-001 activated endogenous Nrf2 and caused nuclear accumulation in SCC VII cells.

Next, we investigated RRx-001-induced activation of endogenous Nrf2 in SCC VII tumors *in vivo*. Mice bearing SCC VII tumors were treated with a single intravenous dose of 10 mg/kg RRx-001. Tumors were collected 24 or 48 h later and cytoplasmic and nuclear proteins were isolated for immunoblot assay. As shown in Figure 1B, the intensity of both cytoplasmic and nuclear Nrf2 proteins was much higher in RRx-001-treated tumors compared to that from control tumors without RRx-001 treatment (CTL). The cytoplasmic Nrf2 proteins were increased by 2.0- and 2.4- fold in tumors at 24 h and 48 h, respectively, after a single dose of 10 mg/kg RRx-001 compared to control tumor without RRx-001 treatment. The nuclear Nrf2 level also increased to ~ 1.6- and 2.3- fold at 24 h and 48 h, respectively, after 10 mg/kg RRx-001 treatment. These results indicate that treatment with RRx-001 not only facilitated nuclear translocation of Nrf2, but also upregulated endogenous Nrf2 expression in SCC VII tumors in mice.

To verify the effect of RRx-001 on Nrf2 activation, we assayed the expression of Nrf2 downstream antioxidant enzymes HO-1 and NQO1 in RRx-001 treated cells *in vitro* and *in vivo* in tumor-bearing mice. SCC VII cells were exposed to 0, 2, or 5 μM of RRx-001 in growth medium for 8 h or 24 h, and whole cell lysates were used to assay the HO-1 and NQO1 enzyme levels by Western blot. For the *in vivo* study, mice bearing SCC VII tumors were injected intravenously with one dose of either solvent or 10 mg/kg RRx-001, and ~ 8 h and 24 h later tumors were collected after whole animal perfusion with PBS. Whole tumor homogenates were used to assay the HO-1 and NQO1 protein levels by Western blot. As shown in Figure 1C, the levels of HO-1 and NQO1 in RRx-001 treated cells *in vitro* were significantly increased in a dose- and time-dependent manner. HO-1 enzyme increased by ~2-fold after 8 h exposure, and then returned to the basal level at 24 h. The NQO1 enzyme increased by 2- to 4-fold at 8 h and over 6-fold at 24 h. The levels of HO-1 and NQO1 in RRx-001-treated tumors in mice were also increased by 13% and 20% for HO-1, and 12% and 27% for NQO1 at 8 h and 24 h, respectively, post-RRx-001 injection, compared to solvent control tumors (*p* < 0.05 for all RRx-001-treated tumors compared to control, *n* = 6 per time point) (Figure 1D).

### Effect of RRx-001 in SCC VII cells with knockdown of endogenous Nrf2

**Figure 2:**
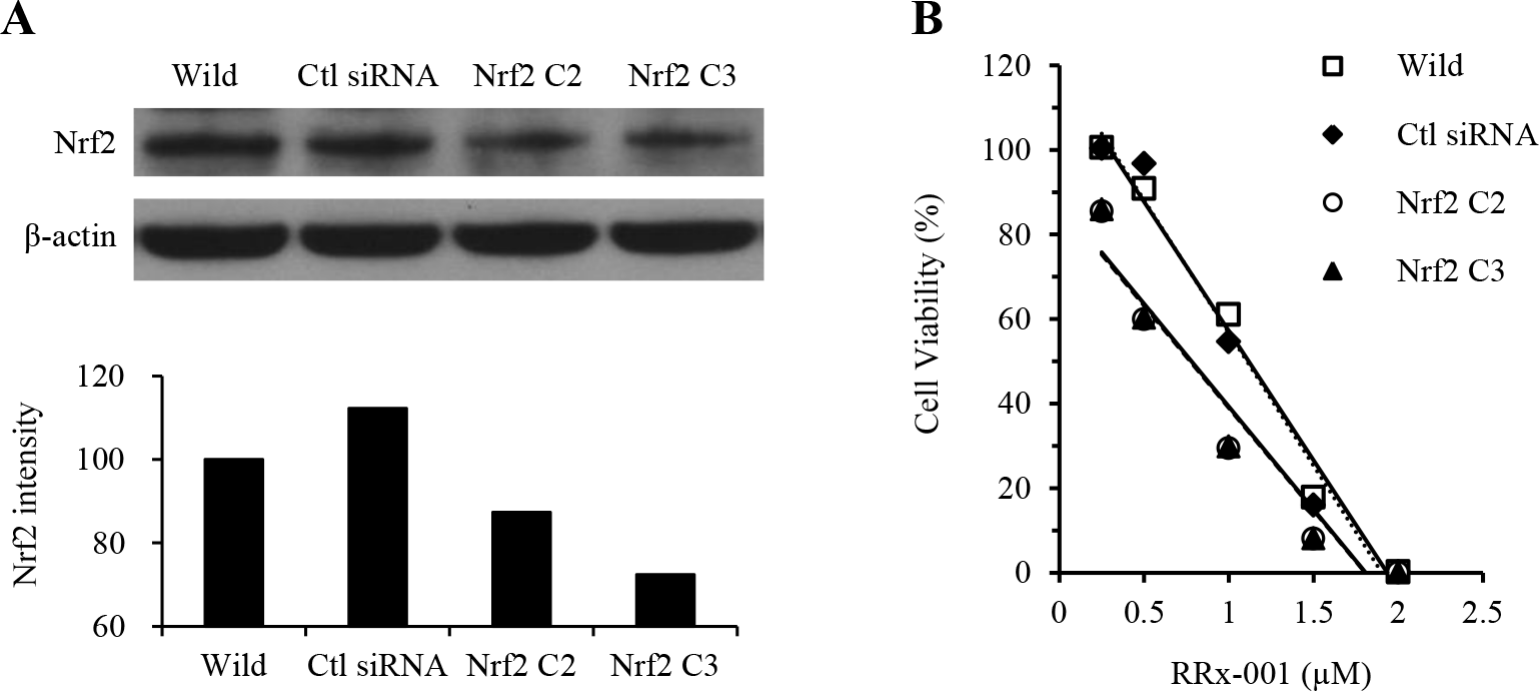
Effect of RRx-001 in SCC VII tumor cells transfected with Nrf2-specific siRNA **A.** Western blot and quantitative graph showing the level of Nrf2 expression in cells transfected with control siRNA and Nrf2-specific siRNA. **B.** Cell viability assay in cells transfected with scrambled and Nrf2-specific siRNA in response to the treatment of different concentrations of RRx-001.

SCC VII cells were transiently transfected with either Nrf2 siRNA or scrambled siRNA, and Nrf2 expression and response to RRx-001 were assayed. As shown in Figure 2A, Nrf2 expression was significantly decreased in cells transfected with Nrf2 siRNA (Nrf2 C2 and Nrf2 C3). The protein level of Nrf2 was decreased by 40% compared to the level in cells transfected with scrambled siRNA (Ctl siRNA). We then analyzed the response of cells to RRx-001 treatment by using a modified MTT cell proliferation assay (WST-8 assay). Cells transfected with Nrf2 siRNA (Nrf2 C2 and Nrf2 C3) were more sensitive to RRx-001 compared to parental cells (wild) or cells transfected with scrambled siRNA (Ctl siRNA), with IC_50_ of 1.09, 1.04, 0.74, and 0.75 μM for parental, scrambled siRNA and Nrf2 siRNA-transfected C2 and C3 cells, respectively (Figure 2B).

### RRx-001-mediated activation of ARE signaling in dual reporter gene transfected cells

**Figure 3:**
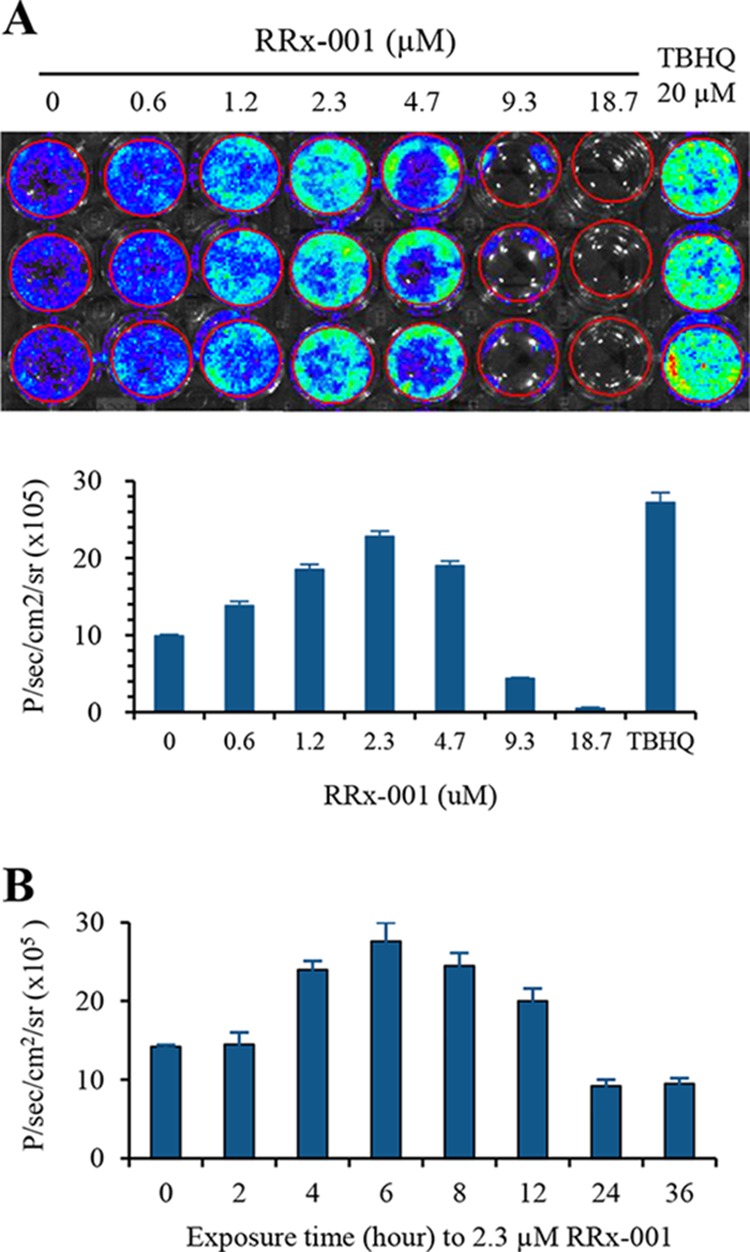
Reporter gene imaging to study the activation of ARE signaling by RRx-001 in SCC VII cells **A.** Optical bioluminescence imaging and quantitative graph of ARE-FLUC signal in SCC VII cells stably expressing ARE-FLUC, 6 h after treatment with different doses of RRx-001. **B.** ARE-FLUC signal measured in SCC VII stable cells at different time points after treating with 2.3 μM of RRx-001.

SCC VII tumor cells were stably co-transfected with ARE-Firefly luciferase (ARE-FLUC) pathway reporter gene and CMV-Renilla luciferase-monomeric red fluorescent protein (RLUC-mRFP). The ARE-FLUC reporter gene was used to measure ARE signaling, while the constitutive RLUC-mRFP signal was used to measure the cell viability in response to RRx-001 treatment. Positive clones were double selected by puromycin and G418, and further enriched by FACS, and verified for the expression of both reporter genes by a dual-luciferase reporter assay system. A single clone of cells co-expressing ARE-FLUC and CMV-RLUC-mRFP was expanded and used for evaluating ARE-FLUC response to RRx-001 treatment. The known Nrf2 activator TBHQ was used as a positive control in the study. The results showed that there was a dose- and time-dependent activation of ARE-FLUC reporter gene by RRx-001, with a maximum activation at 6 h post-treatment with 2.3 μM of RRx-001 (Figure 3). The ARE-FLUC reporter signals gradually decreased at RRx-001 doses of 4.7–18.7 μM, mainly due to the cell killing effect at these high doses of RRx-001 as indicated by decreased CMV-RLUC-mRFP signals. These results indicate that RRx-001 is an effective activator of the Nrf2-ARE pathway.

### Imaging of ARE reporter gene activation in SCC VII tumors in mice

**Figure 4:**
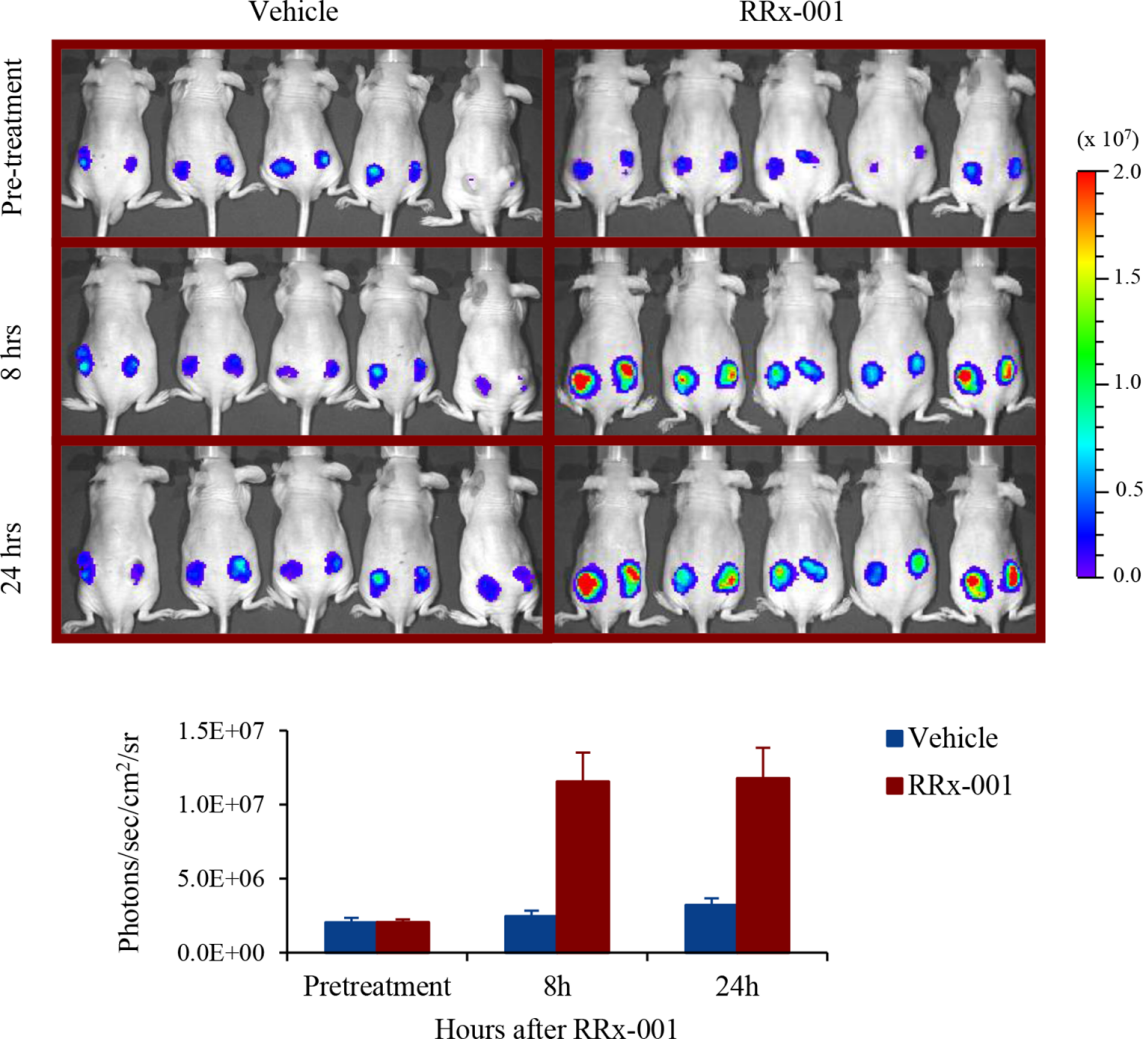
Optical bioluminescence imaging of ARE-FLUC activation by RRx-001 treatment in nude mice bearing SCC VII tumor xenograft **A.** Bioluminescence signal measured 24 h before (Pre-treatment) and 8 h and 24 h after treatment with RRx-001 by IVIS optical bioluminescence imaging *in vivo* in animals. **B.** Quantitative graph showing the bioluminescence signal measured from image shown in (A)

Mice bearing ARE-FLUC/RLUC-mRFP-expressing SCC VII tumors were treated with a single intravenous dose of 10 mg/kg RRx-001 and imaged for ARE-FLUC expression at 24 h before (pre-treatment) and 8 h and 24 h post-RRx-001 injection with a Xenogen IVIS 200 Imaging System. There were 10 mice (total of 20 tumors; 2 tumors per mouse) in each of RRx-001- and vehicle-treated groups. Figure 4 shows representative images of SCC VII tumors in mice with 5 animals/10 tumors for each group. The pre-treatment images were taken 24 h before RRx-001 injection. The quantitation of ARE reporter gene signal was normalized by the RLUC-mRFP signal from the same tumor. There was a significant increase in the ARE-FLUC signals (5-fold higher compared to control) at 8 h following a single dose of RRx-001 treatment, and that was maintained at high levels until 24 h, while there was no obvious change in the ARE-FLUC signals in the vehicle control-treated group. In a separate study, we treated mice with a lower dose of 5 mg/kg RRx-001 and found that the level of activation of ARE-FLUC signals was not significantly different from 10 mg/kg RRx-001 (Supplemental Figure 1).

### Correlation of ARE activity and tumor response to RRx-001 treatment

Many studies have reported that the overexpression of Nrf2 and its downstream genes in cancer cells provides a survival benefit and growth advantage [7]. The Nrf2-ARE signaling pathway is also involved in acquisition of resistance against anticancer therapies through activation of antioxidant genes and suppression of ROS accumulation and neutralization of electrophiles [7, 15–17]. We first evaluated the effect of co-expression of ARE-FLUC and CMV-RLUC-mRFP reporters on SCC VII tumor growth. Mice were subcutaneously inoculated with 5 × 10^5^ cells of the parental and ARE-FLUC/CMV-RLUC-mRFP expressing SCC VII cells, and the tumor take rate and tumor size were monitored daily for 14 days. Results showed that the tumor take rates in syngeneic mice were 95% (19 tumors of 20 implanted) for both the parental and the ARE reporter-expressing tumor implants. The ARE-FLUC/CMV-RLUC-mRFP expressing tumors grew as aggressively as the parental tumors in mice with a tumor volume quadrupling time (TVQT, 4 times its pretreatment volume) of approximately 3.0 ± 0.3 and 3.5 ± 0.7 days for the parental and the ARE-FLUC/CMV-RLUC-mRFP tumors, respectively (Supplemental Figure 2). There was no statistical difference in TVQT between the parental and the ARE-expressing tumors (*n* = 8, *P* = 0.09). The result indicates that the ARE reporter transfection did not significantly alter the SCC VII tumor growth pattern.

**Figure 5:**
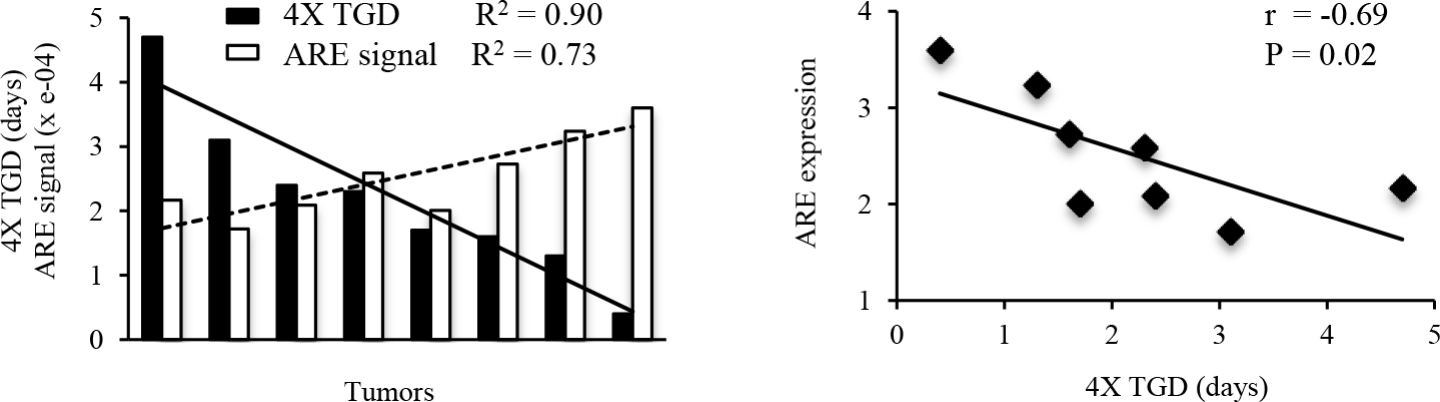
Correlation of pre-treatment ARE expression vs. tumor response to RRx-001 treatment

Next, we studied the tumor response to RRx-001 treatment and analyzed the correlation between the tumor response and the ARE reporter gene expression before and after RRx-001 treatment. Mice were subcutaneously inoculated with 5 × 10^5^ ARE-FLUC/CMV-RLUC-mRFP expressing SCC VII cells. Two tumors per animal were inoculated on the left and right lower flank. Ten days after tumor inoculation, mice with tumors of average size of 150 mm^3^ were selected and randomized into two groups with 8 tumors per group: 1) vehicle control and 2) RRx-001 treatment group. Mice were imaged for ARE-FLUC expression 24 h before and after RRx-001 administration. Results showed that a single dose of 10 mg/kg RRx-001 inhibited tumor growth and produced a tumor volume quadrupling time (TVQT) of 5.7 ± 1.3 days compared to 3.3 ± 0.7 days of vehicle control group (*P* < 0.05). The linear regression analysis of the tumor growth delay (4X TGD, i.e. the difference between the TVQT of RRx-001-treated tumors compared to that of untreated control tumors) showed that the 4X TGD of each tumor was negatively correlated to its pre-treatment level of the ARE signal (Figure 5; *R^2^* = −0.69, *P* = 0.02). A relatively low level of the pre-treatment ARE-FLUC signal correlated with improved response to RRx-001 compared to tumors with no pre-treatment signal; this same pattern of correlation occurred even among individual tumors within the same host mouse. The correlation analysis also showed that there were no correlations between post-treatment ARE-FLUC signals at 8 h – 24 h and the tumor response to RRx-001 treatment (*R^2^* = 0.2 – 0.4, *P* > 0.05). Taken together, the data suggest that the pre-treatment ARE expression, reflecting the basal level of Nrf2 activity, is a biomarker for early prediction of therapeutic response to pro-oxidant RRx-001 treatment.

## DISCUSSION

RRx-001 is a small molecular pan-epigenetic agent with ROS/RNS-generating properties that targets hypoxic tumor cells [11–13]. Antitumor activity has been demonstrated both in preclinical models and in patients with heavily pretreated refractory solid tumors [11, 14]. Apart from epigenetic alterations, RRx-001 acts via pleiotropic mechanisms including redox signaling and redox-induced dysregulation of many different signal pathways such as Nrf2, p53, PARP cleavage, HIF1 alpha, and G6PD activity [18]. RRx-001 also triggers p53 and p21 activity in response to double-stranded DNA breaks as well as deregulates cancer cellular energetics and metabolism [13]. In this study, we explored the impact of RRx-001 on Nrf2 activation in SCC VII tumor cells harboring the ARE-FLUC reporter vector. When ARE-FLUC-expressing SCC VII cells were treated with RRx-001, activated ARE-FLUC signal was clearly detected. The ARE-FLUC signal after exposure to 2.3 μM RRx-001 was 2.3-fold higher than observed in untreated control cells, and equal to 80% of the signal achieved from cells treated with 20 μM of TBHQ, a known Nrf2 activator.

Nrf2 is a transcription factor that controls the expression of a set of phase II detoxifying enzymes such as NQO1, HO-1, GCLC, GST, UGT, and Mrp. Nrf2 signaling itself is regulated by a variety of mechanisms from the transcriptional control of the Nrf2 gene to activation of ARE by the Nrf2 complex. The Nrf2-Keap1 complex is the most important regulator of Nrf2 nuclear translocation through proteasomal degradation [19]. Keap1, an E3-ubiquitin ligase, targets Nrf2 for ubiquitin-dependent degradation, and is the major mechanism by which Nrf2 nuclear levels are tightly controlled in cells. In addition to Keap1, Nrf2 degradation is also controlled by glycogen synthase kinase-3 (GSK-3)-mediated phosphorylation of two of its serine residues in the sequence DSGISL, which allows β-transducin repeat-containing protein (β-TrCP) to ubiquitinate Nrf2 and promote its degradation [20]. In parallel to these proteins, many other proteins are identified as Nrf2 stabilizers either by directly binding with Nrf2, such as p21 ^Cip1/WAF1^ [21], BRCA1 [22–23] and DJ-1 [24], or through indirect binding with Keap1 [25]. In oxidative stress, Nrf2 is phosphorylated and trafficked into the nucleus where it recruits its client protein small-maf and binds to antioxidant response elements (ARE), leading to the coordinated induction of antioxidant genes. Similarly, through the release of ROS and RNS, RRx-001 activates Nrf2 with the subsequent induction of downstream target gene expression.

Nrf2 activity has been labeled a “double-edged sword” with both anti- and pro-tumorigenic properties, which can be both beneficial and deleterious: on the one hand, Nrf2 protects normal cells from oxidative stress and confers protection against tumorigenesis, while, on the other hand, it promotes ROS detoxification and tumor cell proliferation [7]. In general, the Nrf2-Keap1 pathway protects the cell and exerts a beneficial effect, delaying or preventing the onset of diseases like atherosclerosis, Alzheimer's disease, and rheumatoid arthritis [2]. However, the dark side of Nrf2 is that it also contributes to chemo- and radio-resistance of human cancers [15–17], since activation of the Nrf2-Keap1 pathway by anticancer therapeutics increases the intracellular antioxidant capacity and therefore not only protects cancer cells but may also accelerate their proliferation. In this context, Nrf2 inhibitors may help to overcome treatment resistance. Hence anticancer therapies may be made more effective if the mechanisms by which Nrf2 activators and/or inhibitors mediate the transition from anti- to pro-apoptotic effects are better understood. As demonstrated here, RRx-001 activates Nrf2-ARE signaling while exerting cytotoxicity by oxidative damage; however its effect in tumors with higher basal levels of activated Nrf2-ARE signaling is reduced as compared to tumors with lower activation.

Nrf2 regulates the production of mitochondrial and cytosolic ROS through NADPH oxidase [26] and therefore knockdown of Nrf2 provokes ROS accumulation and ultimately induces lethal DNA damage. As we reported here, genetic knockdown of the endogenous Nrf2 expression by Nrf2-specific siRNA increased the sensitivity of cancer cells to RRx-001. This result clearly suggests that in combination with Nrf2 inhibitors the anti-cancer properties of RRx-001 will be enhanced, while RRx-001-induced activation of Nrf2 may have beneficial effects on ROS-mediated diseases like diabetes, rheumatoid arthritis and atherosclerosis. In fact, a number of chemically diverse Nrf2 activators have been identified and preclinically evaluated for therapeutic properties. Activation of Nrf2 by sulforaphane reduced hyperglycemia-induced stimulation of the hexosamine and PKC pathways and increased excretion of the glycating agent, methylglyoxal [27]. Cinnamic aldehyde, caffeic acid phenethylester and bardoxolone methyl, which similarly activate Nrf2, are under investigation for treatment of diabetes complications [28, 29]. Beside these chemical compounds, varieties of natural compounds have also been identified as Nrf2 activators [30, 31]. However, all of the above activators, unlike RRx-001, lack apoptosis-inducing properties, and further research is needed to determine their therapeutic potential in other major diseases.

In summary, molecular imaging of ARE-FLUC and RLUC-mRFP co-expressing tumors *in vitro* and in tumor-bearing mice reveals that RRx-001 is a potent activator of the Nrf2-ARE signaling pathway via ROS/RNS generation. The level of the pretreatment signal of ARE-FLUC in tumors is inversely correlated with the tumor response to RRx-001 treatment. Inhibition of endogenous Nrf2 expression by Nrf2-specific siRNA increases the cellular sensitivity to RRx-001 treatment. These results suggest that the basal level of Nrf2 and/or its downstream gene expression and/or genes expressed under ARE may serve as a biomarker for predicting therapeutic response of cancers to RRx-001 treatment, and for selection and stratification of cancer patients who would be most likely to respond and benefit from RRx-001 therapy. Furthermore, the combined use of RRx-001 and Nrf2 inhibitors may enhance the anticancer efficacy and therefore this approach requires further study.

## MATERIALS AND METHODS

### Materials

RRx-001 was obtained from ATK Aerospace Systems [10]. The synthesis and characterization of RRx-001 is reported in detail elsewhere [8–12]. For *in vitro* cell culture experiments, RRx-001 was dissolved in DMSO and then diluted with growth medium with a final concentration of DMSO at < 0.05%. For animal experiments, RRx-001 formulation was prepared by dissolving 10 mg RRx-001 in 0.5 mL DMA-PEG 400 (1:2) and then diluting with double distilled water to obtain a 2 mg/mL solution for injection.

The pcDNA-ARE-FLUC and pcDNA-CMV-RLUC-mRFP vectors were constructed in our lab. Nrf2-specific small interfering RNA (Nrf2-siRNA, sc-37049), scrambled siRNA (sc-37007), transfection reagents (sc-29528 and sc-36868), and antibodies against Nrf2 (sc-13032), HO-1 (sc-10789), NQO1 (sc-393736), Δ-actin (sc-130656) and lamin-B (sc-365962) were purchased from Santa Cruz Biotechnology, Santa Cruz, CA.

### Cell culture and transfection

The SCC VII murine squamous cell carcinoma cells (32) were grown and maintained in DMEM medium (Invitrogen, Carlsbad, CA) supplemented with 10% fetal calf serum, 100 units/ml penicillin, and 100 μg/mL streptomycin in a 37°C humidified incubator with a mixture of 95% air and 5% CO_2_. The identity of cells has regularly been confirmed throughout the course of the studies by observation of the growth pattern and cell morphology *in vitro* and *in vivo*. All experiments were performed on exponentially growing cells with cell population doubling times of approximately 20 h.

To silence the expression of endogenous Nrf2, SCC VII cells were transiently transfected with Nrf2-specific small interfering RNA (Nrf2-siRNA) by following the manufacturer's protocol. SCC VII cells were also transfected with a scrambled siRNA as a non-specific control. Briefly, SCC VII cells were seeded at a density of 1.0 × 10^6^ cells/60-mm dish and transfected with either 120 pmol of Nrf2 siRNA or scrambled siRNA in siRNA transfection reagent-containing medium for 24 h at 37°C. The siRNA-containing medium was then replaced with normal growth medium and incubated for an additional 24 h. Nrf2 expression and response to RRx-001 treatment were then assayed using Western blot and WST-8 assay (Dojindo Molecular Technologies, Santa Clara, CA) as previously reported [11].

To create a stable cell line co-expressing ARE-Firefly luciferase (ARE-FLUC) and CMV-Renilla luciferase-monomeric red fluorescent protein (RLUC-mRFP) fusion proteins, cells at 80% confluency in 100-mm plates were co-transfected with 10 μg each of pcPUR-ARE-FLUC and pcDNA-CMV-RLUC-mRFP plasmids by lipofectamine-mediated transfection (Supplemental Figure 3). Single clones of cells co-expressing both the reporter genes were double selected by puromycin and G418, and a colony was picked after bioluminescence imaging and sorted twice by FACS in RFP window. The expanded cells were evaluated by dual-luciferase reporter assay for FLUC and RLUC reporter activities *in vitro*.

### *In vitro* dual-luciferase assay

A dual-luciferase reporter assay was performed to verify the expression of FLUC and RLUC in transfected SCC VII tumor cells. Cells (1 × 10^6^) were lysed in 200 μl of passive lysis buffer (Promega, Madison, WI) by gently shaking for 10 min at room temperature, and the whole cell lysate was centrifuged at 10, 000 rpm for 5 min. The cleared supernatant of 20 μl was mixed with 100 μl of LARII solution (Promega) and measured for 10 seconds on a GloMax-20/20-luminometer (Promega). Similarly, a 20 μl aliquot of lysate was mixed with 1 μg of coelenterazine in 100 μl of PBS for RLUC signal measurement by luminometer. The total protein content of each sample was used to normalize the results. The cells were also tested for the ARE-FLUC response by inducing with the known Nrf2-activator tert-butylhydroquinone (TBHQ) and with RRx-001 in various concentrations at different time points.

### Western blot analysis

After exposure to RRx-001, cells were washed twice with cold PBS and lysed in RIPA buffer or NE-PER nuclear and cytoplasmic extraction buffer (Pierce Biotech, Rockford, IL) for extraction and separation of the cytosolic and nuclear proteins. For tumor samples, tumor-bearing mice were euthanized in a CO_2_ chamber at designated time points after RRx-001 treatment and perfused with 50 ml PBS to remove blood cells. Tumors were homogenized in 20 mM HEPES buffer (containing 1 mM EGTA, 210 mM mannitol and 70 mM sucrose), centrifuged and the supernatants were used for Western blot. The protein contents were quantified using a Bio-Rad protein assay kit (Bio-Rad, Hercules, CA). Samples containing equal amounts of total protein (20 μg) were resolved in 10% SDS-PAGE gel and transferred onto PVDF membrane. The membrane was blocked with 5% non-fat milk and probed with primary antibody and HRP-conjugated secondary antibody (Santa Cruz Biotechnology). The immunoreactive proteins were detected with ECL plus chemiluminescence detection reagents (Amersham Biosciences, GE Life Sciences, Pittsburgh, PA), and quantified by ImageJ program (v1.47, NIH). The Western blot analyses were run at least twice, unless otherwise specifically indicated.

### Tumor model and therapy

Nude mice, male, 7–8 weeks old and 20–25 grams in body weight, were purchased from Charles River Laboratories. Mice were acclimated under specific pathogen-free conditions in the Veterinary Service Center of Stanford University animal facilities for 3–5 days before starting each experiment, and sterilized food and water were available *ad libitum*. Mice were injected subcutaneously in the left and right lower flank with 5 × 10^5^ SCC VII tumor cells stably co-expressing ARE-FLUC and CMV-RLUC-mRFP in 0.05 ml Hank's solution. Two tumors were implanted per mouse. When tumors reached an average size of 150 mm^3^ (10 days after implantation), mice were randomly assigned to the treatment groups and imaged. RRx-001 was injected i.v. at doses as specified in each experiment. The tumor size was measured with calipers before treatment, and three times a week thereafter until the tumor volume reached at least 4 times (4 ×) the pre-treatment volume. The tumor volume was calculated using the formula: tumor volume = π/6 × length × width^2^. The tumor volume quadrupling time (TVQT, 4 ×)) was determined by a best-fit regression analysis. The tumor growth delay (TGD) is the difference between the TVQT of treated tumors compared to that of untreated control tumors. Both the TVQT and TGD were calculated for each individual animal, and then averaged for each group. The data are presented as percent (%) of the pretreatment volume on day 0. Body weight of animals was measured three times a week. The animal experiments described herein were approved by the Stanford University Administrative Panel for Laboratory Animal Care.

### Bioluminescence imaging (BLI)

A Xenogen IVIS 200 Imaging System was used in this study. Tumors were imaged for basal FLUC, RLUC, and mRFP signals 24 h before administration of RRx-001. Following injection with RRx-001, mice were imaged for RLUC signal by intravenous injection of 50 μg of coelenterazine at 8 and 24 h post-RRx-001 treatment. Similarly, mice were imaged for FLUC signal 6 h after RLUC imaging by intraperitoneal injection of 3 mg of D-Luciferin in 100 μl PBS. The animals injected with vehicle (without RRx-001) served as the untreated control. The BLI signals over the region of interest (ROI) were quantified by Living Image software (Caliper Life Sciences, Alameda, CA). The BLI signals in RRx-001-treated tumors were quantitated and compared with the BLI signals of untreated control tumors. The mRFP signal was collected at all imaging sessions by excitation at 580 nm/emission at 610 nm.

### Statistics

Data were statistically analyzed using a two-tailed Student's *t*-test. The correlation between the tumor response to RRx-001 treatment and the level of ARE signal was analyzed by a linear regression analysis.

## SUPPLEMENTARY FIGURES


