# Supplementary material for: Nrf2 activity as a potential biomarker for the pan-epigenetic anticancer agent, RRx-001
Source: Oncotarget. 2015 Jun 4;6(25):21547–56. doi: 10.18632/oncotarget.4249 (PMC4673285; doi:10.18632/oncotarget.4249)
Supplement: Supplementary file 1 [file oncotarget-06-21547-s001.pdf]

SUPPLEMENTARY FIGURES

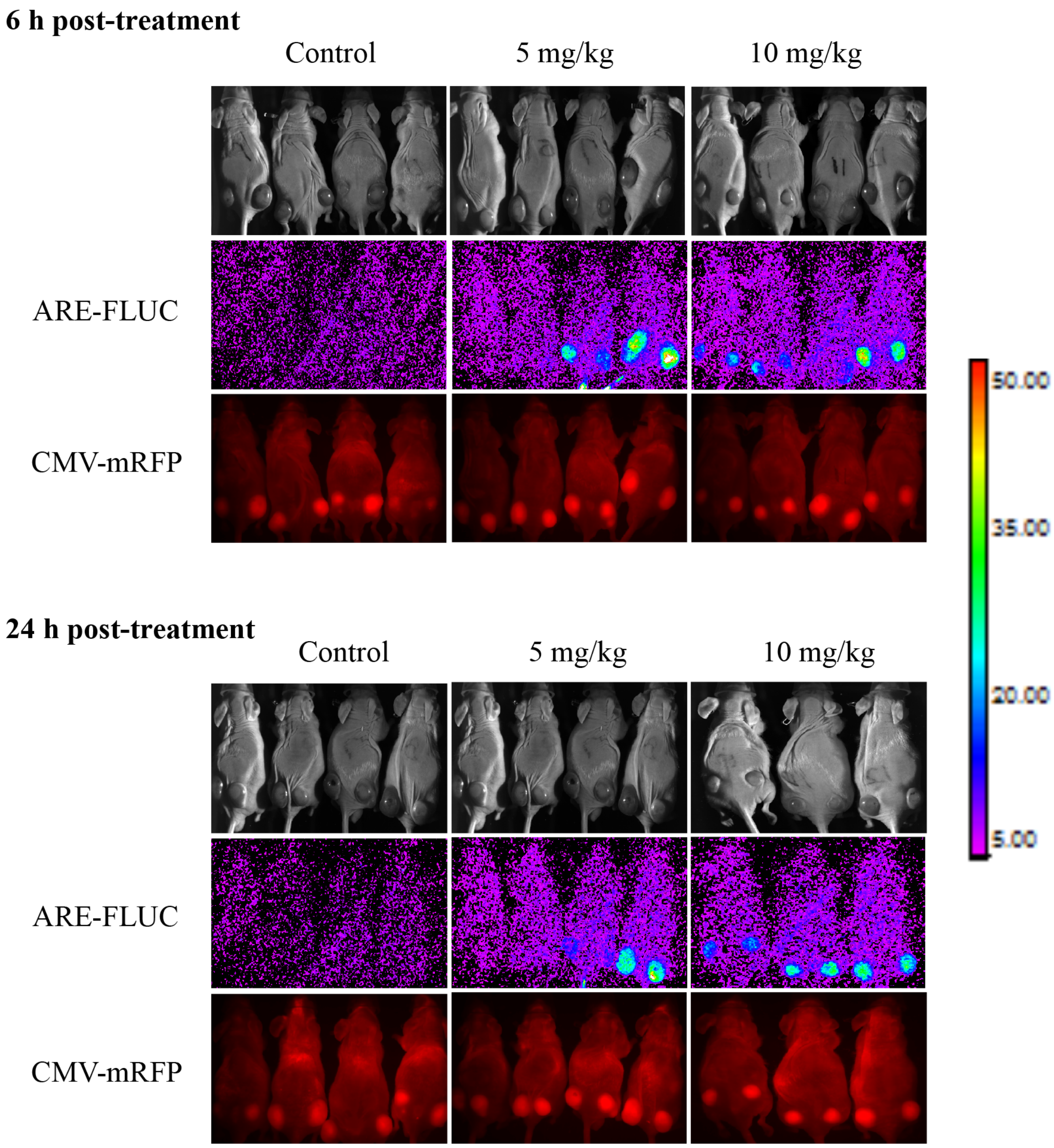

Supplementary Figure S1: Optical bioluminescence imaging of ARE-FLUC activation by RRx-001 in nude mice bearing SCC VII tumors. Mice were treated with one dose of 5 mg/kg or 10 mg/kg RRx-001 and imaged 6 h and 24 h later. The white light image was taken immediately before BLI imaging showing the mice with subcutaneous tumors. The CMV-mRFP images show the tumor size.

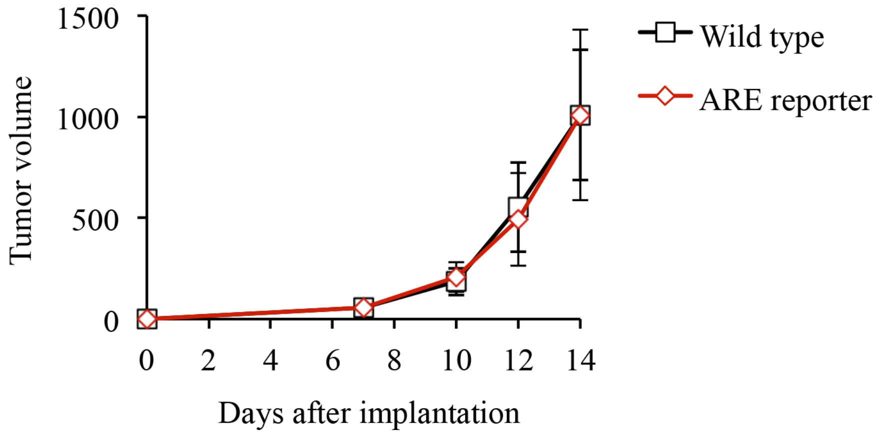

Supplementary Figure S2: Graph showing SCC VII tumor growth in mice. ARE-FLUC/RLUC-mRFP transfection did not alter the tumor growth pattern in mice compared to the wild type parental tumors.

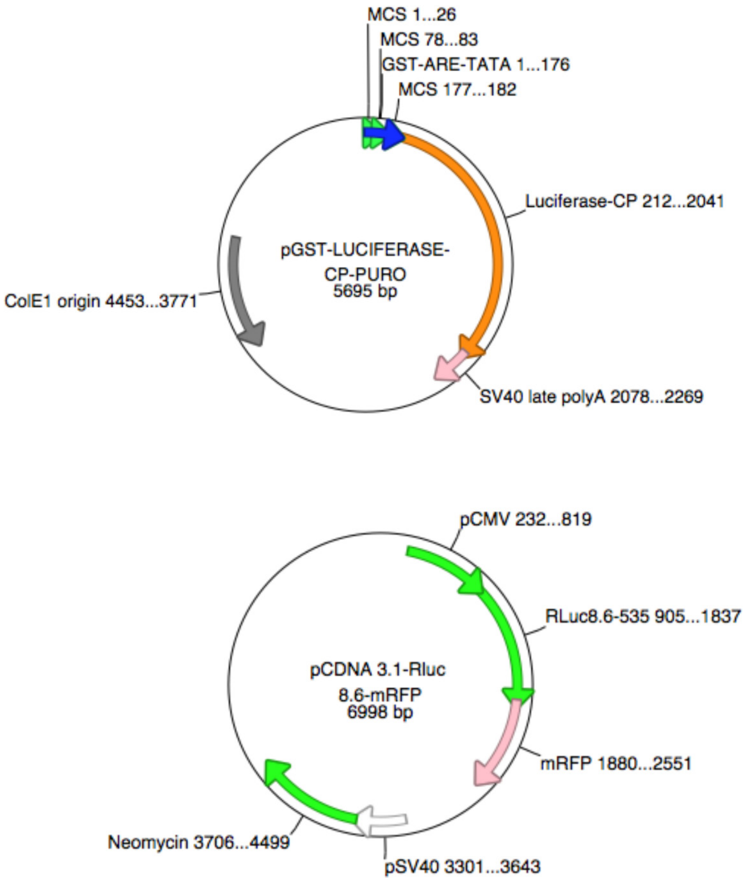

Supplementary Figure S3: Vector maps of pcDNA-ARE-FLUC and pcDNA-CMV-RLUC-RFP.
